# Physiological indicators of emotional arousal related to ANS activity in response to associative cards for psychotherapeutic PTSD treatment

**DOI:** 10.3389/fpsyt.2022.933692

**Published:** 2022-11-07

**Authors:** Sivan Raz, Mooli Lahad

**Affiliations:** ^1^Department of Psychology, Tel-Hai College, Upper Galilee, Israel; ^2^Department of Behavioral Sciences, Max Stern Yezreel Valley College, Emek Yezreel, Israel

**Keywords:** PTSD, therapy, SEE FAR CBT, COPE cards, heart rate variability, blood pressure

## Abstract

SEE FAR CBT is an integrative treatment protocol for PTSD and anxiety disorders which combines CBT, body-mind (somatic experience) and imagery-based (fantastic reality; FR) methods. FR is introduced using associative therapeutic cards (COPE cards) to represent both “a pleasant/safe place” and the re-narrating process of the traumatic story. Although some preliminary evidence exists regarding the impact of COPE cards integration in psychotherapy, further validation is needed as to whether these cards can induce distinct arousal-affective states in the observer. The aim of this study was to examine whether exposure to COPE cards evoke different emotional-psychophysiological states using objective physiological measures reflecting autonomic nervous system responses; hence, to further validate its use as a potentially effective tool within the context of SEE FAR CBT therapeutic process. Ninety-five healthy under-graduate participants were first exposed to high-arousal, negatively-valenced cards and asked to put themselves in a state of emotional/physical arousal. Afterwards, they were exposed to low-arousal, positively-valenced cards and were asked to try to calm and relax to the best of their ability. Heart rate, blood pressure and heart rate variability (HRV) were measured at baseline, at the arousal phase and finally at the relaxation phase. It was found that exposure to arousing negative cards resulted in significant increase in blood pressure and a decrease in HRV, while exposure to relaxing positive cards resulted in significant decrease in blood pressure and an increase in HRV. These findings support the efficacy and utility of associative COPE cards in affecting psychophysiological arousal.

## Introduction

Posttraumatic stress disorder (PTSD) is a chronic mental health disorder that may develop after experiencing or witnessing a traumatic event, such as military combat, terror attack, natural disaster, serious injury, sexual assault, or sudden death of a loved one. PTSD is associated with a wide range of significant problems including occupational difficulties, social dysfunction, difficulties in daily life activities and physical health problems (1–3). PTSD symptoms include intrusion symptoms (re-experiencing the traumatic event), avoidance symptoms (avoiding trauma-related stimuli), negative alterations in mood and cognition and hyperarousal (4).

During the late 20th century there has been major development in the field of psycho-trauma; and psychological interventions from various theoretical perspectives have been found to be effective for chronic PTSD. Trauma-focused treatments such as Prolonged Exposure (PE), Eye Movement Desensitization and Reprocessing (EMDR), Cognitive Processing Therapy (CPT), and Cognitive-Behavioral Therapy (CBT), address memories of the traumatic experience, or feeling and thoughts associated with it, in a direct and explicit manner. Non-trauma-focused treatments such as Stress Inoculation Training (SIT), relaxation techniques, present-centered therapy, and interpersonal therapy, aim to relieve PTSD symptoms without directly targeting the traumatic event-related memories, feelings, and thoughts (1, 5, 6).

The trauma-focused CBT approach has gained a large clinical and experimental evidence base supporting its effectiveness in treating PTSD (3, 7, 8).

SEE FAR CBT is an integrative treatment protocol for PTSD and anxiety disorders which combines CBT, body-mind (somatic experience; SE) and imagery-based (fantastic reality; FR) methods [for detailed explanation, see (9)]. In a study by Lahad et al. (10), adult PTSD patients, divided into eye movement desensitization and reprocessing (EMDR) and SEE FAR CBT groups, were assessed for traumatic symptoms at three-time intervals (pre-treatment, post-treatment and 1-year follow-up). While both EMDR and SEE FAR CBT resulted in significant decreases in trauma symptoms, showing effective alleviation of trauma severity over time, they didn't differ from each other in terms of treatment efficacy during any of the assessment time points. In another study, Lahad et al. (11) reported that SEE FAR CBT was significantly more effective at reducing PTSD symptoms immediately after the treatment as well as at 6- months follow up assessment, including lowering somatic complaints, intrusion, arousal, and avoidance behavior, compared to school-based intervention.

The “somatic experience” component of the SEE FAR CBT focuses on the “body memory” or “physiological memory.” This therapeutic approach is based on patient's ability to notice, explore, and report subjective bodily sensations to allow for physiological energy discharge and reducing physiological and mental stress (10, 12). The FR component is a theoretical construct that refer to the ability to transcend into a fantastic space where people may feel safe and secure and where they can reconstruct and deal with the traumatic story (9, 10, 13). In practice, FR is introduced using associative therapeutic cards [COPE cards; (14–16)]. The goal of these cards is to recreate a story that is represented by the cards the client chooses at his discretion. Using the COPE cards as an “externalization” of, or “distancing” from the internally haunting images, allows patients to take the position of the observer in their own drama, thus giving them a sense of control and manageability over the traumatic incident. FR allows to make use of the “as if space,” a fantastic space where all the IFs are possible, by adding “I wish cards” to the story, thus, editing the story, observe options and “defrizzing” the otherwise frozen-story (10, 11, 16).

Cohen et al. (17) have established standardized norms for affective ratings of the COPE cards. Healthy participants were asked to rate their subjective affective state following exposure to each of the cards. Results indicated that the cards produced significantly different affective states (in terms of arousal, valence, and dominance) among the participants. This study was the first to present the subjectively, self-reported, affective states evoked by therapeutic COPE cards. Authors noted however that in order to enhance validity of subjective affective responses, future research should include implementing objective physiological measures.

The aim of the present study was to expand on the initial study by Cohen et al. (17) by examining whether COPE cards exposure may indeed evoke different emotional-psychophysiological states, using objective measures reflecting autonomic nervous system (ANS) responses such as heart rate, blood pressure, and heart rate variability (HRV); and hence to further validate its use as a potentially effective tool within the context of SEE FAR CBT therapeutic process as well as in more general experimental settings.

Emotions experienced by humans while interacting with their environment are associated with varying degrees of physiological arousal generated by the ANS (18). The two complementary branches of the ANS, i.e., the excitatory sympathetic and the inhibitory parasympathetic nervous system, often interact antagonistically to produce varying degrees of arousal depending on the situation. Activity of the sympathetic nervous system which becomes dominant during physical or psychological stress, produces elevated physiological arousal to aid in adapting to the challenge. Heart rate (pulse; number of heart beats per minute) is a common physiological indicator of transitory, short-term arousal levels. Measures of heart rate deviation- from- baseline have been associated with emotional states such as fear, anxiety, and excitement (19, 20). During relatively safe, stable, non-stressful situations, the parasympathetic nervous system becomes dominant, maintaining lower degrees of physiological arousal and decreased heart rate (19). Blood pressure is another widely acceptable physiological indicator of emotional state and psychophysiological arousal. In health and behavioral sciences, it is often included as an indicator of emotion regulation effectiveness (21, 22).

Heart rate variability (HRV) is the fluctuation in the time intervals between adjacent heartbeats. HRV is generated by heart-brain interactions and dynamic non-linear ANS processes; it is a non-invasive electrocardiographic measure of the continuous dynamic interplay between sympathetic and parasympathetic influences on heart rate that index neurocardiac function and represents the heart ability to respond to a variety of environmental and physiological stimuli (19, 23, 24). Short-term recordings of HRV distinguish two main spectral components: a high-frequency component (ranging between 0.15 and 0.40 Hz) considered a marker of parasympathetic control, and a low-frequency component (ranging between 0.04 and 0.15 Hz) that may be produced by both the parasympathetic and sympathetic branches (short-term recordings, as done in this study, may fail to detect very low frequency oscillations that mainly reflect sympathetic activity) (24, 25). The effectiveness of emotion regulation is critically dependent on an individual's ability to adjust physiological arousal on a moment-to moment basis. ANS flexibility allows for rapid generation or alteration of physiological and emotional responses in accordance with situational demands; while ANS rigidity leads to a decreased capacity to generate or modulate physiological and emotional states in synchrony with environmental changes. HRV yields information about ANS flexibility, hence represents the capacity for regulated emotional responding (19). HRV is sensitive to changes in ANS activity associated with stress; high levels of HRV are associated with generally more regulated emotional responding, greater emotional wellbeing, and lower levels of anxiety and worry (26). Low levels of HRV are associated with impaired regulatory and homeostatic ANS functions, which reduce the ability to cope with external and internal stressors (23). Decreased high frequency power (i.e., decreased parasympathetic control) has been correlated with stress, panic, anxiety, and worry (24).

The set of emotional COPE cards is relatively new, and although it has been used in clinical practice for several years, it has not yet been objectively researched. So far, the evidence regarding the effect of card exposure on patients' psychophysiological state and level of arousal is largely based on clinicians' intuitions and subjective assessments. If the cards are to be employed as a therapeutic tool or as an experimental tool in the field of emotion/psychophysiological arousal, there is a need to establish more controlled, objective, statistically supported scientific evidence as to their effects. To be clear, the present study was not intended to examine the therapeutic effect of the cards by performing an actual intervention program, but rather to examine their effectiveness in inducing different states of emotional arousal (which is fundamental to the therapeutic process). We aimed to validate the cards as a tool that may elicit distinct measurable psychophysiological responses, not to validate the effectiveness of the SEE FAR CBT treatment. Therefore, this study included healthy participants and not PTSD patients.

We hypothesized that exposure to different COPE cards would be reflected in measurable changes in physiological indicators of emotional arousal related to ANS responses. More specifically, compared to baseline, we expected higher levels of heart rate and blood pressure and lower levels of HRV in response to arousing negatively-valenced cards; followed by decreased heart rate and blood pressure and increased HRV in response to relaxing positively-valenced cards.

## Method

### Participants

Sample included 95 undergraduate students, 36 men and 59 women, ranging between 21 and 31 years of age (M = 24.98 ± 2.19). None of the participants had prior history of neurological or psychiatric disorders or any kind of chronic physical illness/medical condition. None of them were taking medications at the time of testing. Participants were given course credit according to their academic requirements. The study was approved by the Institutional Review Board and is in accordance with the declaration of Helsinki. Written informed consent was obtained from all participants.

### Instruments and measures

#### COPE cards

**Figure 1:**
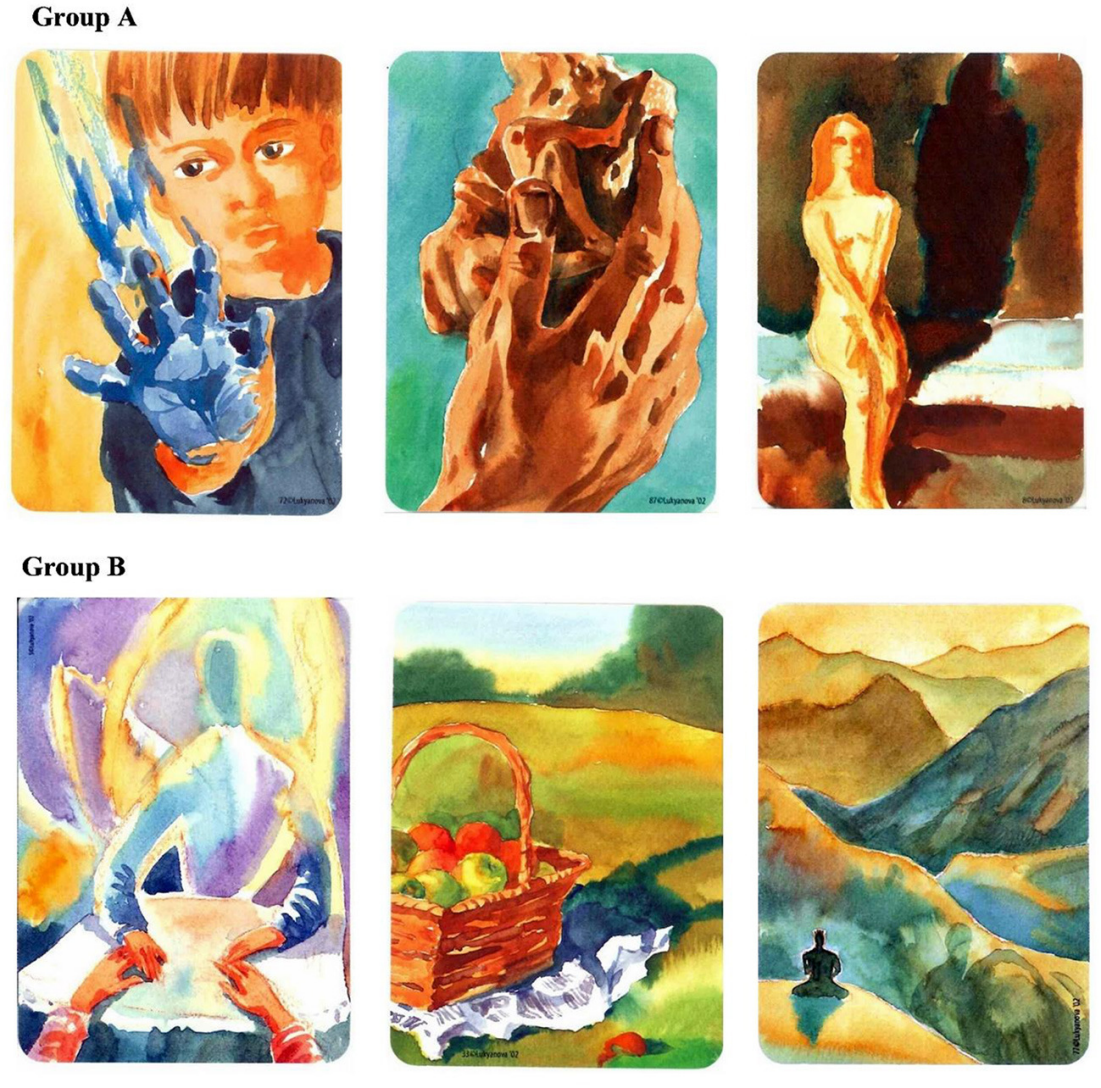
Examples of arousing negative **(A)** and relaxing positive **(B)** COPE cards used in this study.

A package of 88 paintbrush associative images printed on a 7 × 10 cm cards (14). In the current study we used 24 cards chosen based on the Cohen et al. (17) standardized arousal and valence scores; 12 cards presented high-arousal, negatively-valenced images (cards' number: 4, 7, 8, 21, 29, 37, 50, 52, 63, 72, 80, 87), and 12 cards presented low-arousal, relaxing, positively-valenced images (cards' numbers: 1, 2, 3, 5, 32, 33, 35, 49, 56, 62, 66, 77). See Figure 1 for examples of cards of both groups.

#### Physiological measures

Heart rate, blood pressure and heart rate variability (HRV) were used as indicators of psychophysiological arousal and psychophysiological modulation (relaxation) in real time. Heart rate is the number of heart beats per minute. Blood pressure is measured in units of millimeters of mercury (mmHg) using two numbers: Systolic blood pressure reflects the force of blood on the artery walls when the heart contracts, Diastolic blood pressure reflects the force that blood exerts on artery walls when the heart is at rest. Both heart rate and blood pressure were measured using an ambulatory digital arm-cuff pulse and blood pressure monitor [My Check, Artsana S.p.A. (Pic Solution), Italy]. HRV is the variation in the time interval between consecutive heartbeats in milliseconds. It was measured using Elite HRV CorSense^®^ monitor; a one-lead sensor that clips on to the subject's index finger and operated *via* a user-friendly smartphone application. The CorSense^®^ HRV monitor features an array of 3 multi-wavelength LED emitters, 5 large visible spectrum photodetectors, and 1 infrared detector. It relies on its sensor array to provide data collected by a process of photoplethysmography (PPG). PPG is a non-invasive technology that uses a light source and a photodetector at the surface of skin to measure the volumetric variations of blood circulation. While electrocardiogram (ECG) has been considered the most accurate and dominant cardiac monitoring technique, more recent research has confirmed that PPG can provide accurate measures of inter-pulse intervals required for accurate HRV measuring, especially in monitoring healthy individuals [(27–31); “Optimus medica” at https://www.optimusmedica.com/coresense-hrv-monitor-by-elitehrv/]. Readings were collected through the Elite HRV application for mobile devices. The HRV is calculated through receiving the R-R intervals directly from the device. R-R intervals refers to the small changes (milliseconds) in the intervals between successive heartbeats. After performing necessary data cleaning, a Root Mean Square of Successive Differences (RMSSD) calculation is applied to the R-R intervals. Later, a natural log (ln) is applied to RMSSD. The In (RMSSD) is expanded to generate a useful 0 to 100 score to give a value of HRV (32, 33). RMSSD is the standard time domain measurement for detecting ANS activity in short-term measurements (i.e., < 5 min), and it is primarily used to estimate the vagally mediated changes reflected in HRV. RMSSD is correlated with high frequency band power thus may reflect parasympathetic activity (24, 25). HRV, heart rate and blood pressure were measured at three times points as described below (see “Procedure”). Each time, HRV was measured first, and its measurement lasted for 1 min.

### Procedure

Participants were invited to the laboratory session after verifying their compliance with study inclusion criteria. They were instructed to refrain from smoking or drinking alcohol or caffeine for at least 30 min before their arrival at the laboratory. Upon arrival, participants received basic information about the experimental procedure and the aims of study, provided informed consent, and completed a demographic questionnaire. Then the baseline measurement of heart rate, blood pressure and HRV took place (HRV measurement was performed first and lasted 1 min). Participants sat comfortably on an office chair with arms, with their feet flat on the floor, and the cuffed arm resting loosely on a firm surface at heart level. They were instructed to keep still and silent during the readings. Participants then received instructions for self-inducing emotional arousal, after which physiological measures were taken again (Arousal phase). Finally, they were given instructions for modulation (Relaxation phase) and measures were taken for the last time.

#### Instructions for the arousal task

Twelve arousing negative cards were put on a table in front of the participant, and he was asked to choose one of them, take it, look at it carefully and try, to the best of his ability, to put himself in a state of emotional and physical negative arousal/excitement. Experimenter said the following: “Let's assume that discomfort states can be estimated from 0 to 10. A score of 0 means ease, relaxation, pleasure, or a sense of peace and quiet. A score of 10 means feelings of distress, adversity, stress, nervousness, or significant unease. Now, I will lay out cards in front of you, please look at them and look for a card that when you look at it, it makes you feel uncomfortable / stressed / anxious / worried / nervous to a degree of at least 7 on a scale of 0–10. Take it, look at it and try to concentrate / focus on the feelings of unpleasant discomfort that arises. When you feel that you have reached that state, please let me know and I will measure your blood pressure and pulse again. Try to hold these feeling and sensations while I am taking your measures.”

#### Instructions for the modulation/relaxation task

Twelve relaxing positive cards were put on a table in front of the participant, and he was asked to choose one of them and try, to the best of his ability, to relax and to lower the level of emotional and physical arousal. Experimenter said the following: “Now, I will lay out different cards in front of you and this time I will ask you to choose an image that when you look at it, it creates a pleasant, quiet, or safe feeling in you. A feeling of peace and ease. Take the card, look at it, keep the picture in mind and try to deeply feel the pleasantness, relaxation, and calmness that the image evokes in you. Focus on this feeling and let it spread slowly throughout the body. Take a deep breath… let me know when you feel that you have reached a relaxed state, and I will measure your blood pressure and pulse for the last time. keep as calm as you can.”

### Statistical analysis

SPSS-25 was used to perform statistical analyses and Excel was used to produce figures. Four repeated measure ANOVAs were conducted (within-subject design) to examine differences between baseline, arousal phase and relaxation phase in heart rate, systolic blood pressure, diastolic blood pressure and HRV measurements. In cases where Sphericity cannot be assumed, the Greenhouse-Geisser analysis which makes an adjustment to the degrees of freedom was used. Follow-up pairwise comparisons were conducted with the Bonferroni correction to adjust for multiple comparisons. Significance levels are always reported after the Bonferroni correction. Histograms and tests of normality of the standardized residuals at the three time points showed an approximate normal distribution.

## Results

Table 1 presents outcomes of the physiological measurements at the three time points.

**Table 1:** Outcome of measurements (Mean ± SD) at the three time points.

	**Systolic blood pressure**	**Diastolic blood pressure**	**Heart rate**	**HRV**
Baseline	110.07 ± 13.30	68.41 ± 8.91	68.98 ± 9.08	63.23 ± 7.44
Arousal	112.67 ± 13.26	70.80 ± 10.25	69.51 ± 9.59	60.72 ± 4.41
Relaxation	110.51 ± 12.61	69.97 ± 8.65	69.32 ± 9.78	63.14 ± 8.39

### Heart rate

No significant differences in heart rate have emerged between baseline, arousal, and relaxation measurements.

### Systolic blood pressure

**Figure 2:**
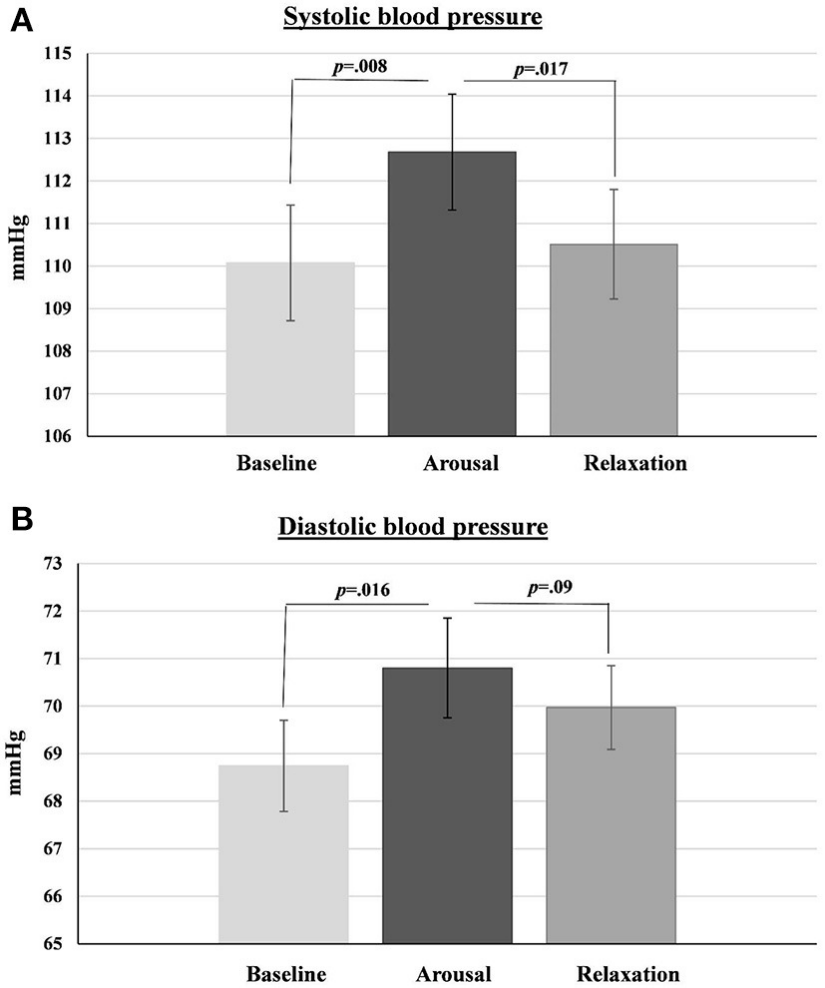
Systolic **(A)** and diastolic **(B)** blood pressure at baseline, arousal phase and relaxation phase. Error bars represent SEM.

Analysis revealed significant differences in systolic blood pressure between measurements at the three time points [*F*_(2,188)_ = 5.96, *p* = 0.003, η*p*^2^ = 0.06, Observed power = 0.88]. Follow-up pairwise comparisons showed that systolic blood pressure was lower at baseline than at the arousal phase (*p* = 0.008; Cohen's d = 0.20), and higher at arousal phase than at relaxation (*p* = 0.017; Cohen's d = 0.17) (Figure 2A).

### Diastolic blood pressure

Analysis revealed significant differences in diastolic blood pressure between measurements at the three time points [*F*_(1.8,167.45)_ = 5.56, *p* = 0.006, η*p*^2^ = 0.06, Observed power = 0.82]. Follow-up comparisons showed that diastolic blood pressure was lower at baseline than at the arousal phase (*p* = 0.016; Cohen's d = 0.24]. Diastolic blood pressure was higher at arousal phase than at relaxation, but this difference didn't reach sufficient statistical significance (*p* = 0.09) (Figure 2B) (One outlier has been removed from this analysis).

### Heart rate variability

**Figure 3:**
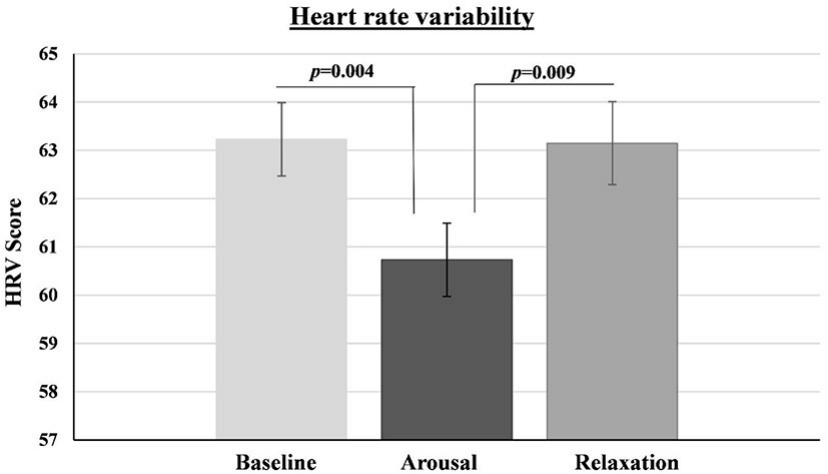
Heart rate variability (HRV) at baseline, arousal phase and relaxation phase. Error bars represent SEM.

Significant differences have been found for HRV between measurements at the three time points [*F*_(2,188)_ = 6.34, *p* = 0.002, η*p*^2^ = 0.06, Observed power = 0.90]. Follow-up comparisons showed that HRV was higher at baseline than at the arousal phase (*p* = 0.004; Cohen's d = 0.34), and lower at arousal phase than at relaxation (*p* = 0.009; Cohen's d = 0.31) (Figure 3)^1^.

## Discussion

The present study aimed at further validating the efficacy and utility of associative COPE cards in affecting psychophysiological arousal by examining whether exposure to these cards is reflected in measurable objective changes in ANS responses. It was found that exposure to arousing negative cards resulted in an increase in blood pressure and a decrease in HRV; probably indicative of decreased parasympathetic activity during unpleasant emotional arousal, while exposure to relaxing positive cards resulted in decrease in blood pressure and an increase in HRV. These findings are in line with studies who examined physiological ANS responses during exposure to emotional pictures taken from the well-established and extensively used international affective picture system [IAPS; (37)] [e.g., (38–42)], which led investigators to suggest that blood pressure and especially HRV could be an objective tools to assess emotional responses and arousal. The similarity between the findings of the IAPS studies and those of the present one, contribute to the validation of the COPE cards as a reliable tool for the purpose of evoking emotional and physical arousal as well as relaxion.

Most approaches in the field of trauma therapy directly refer to invasive thoughts and memories; usually by using words to process the traumatic experiences. However, while it is acknowledged that traumatic experiences include not only verbal cognitive aspects but also non-verbal emotional, sensorial, and physical/somatic phenomena, non-verbal domains such as imagination and creativity have rarely been the center of attention in psychotrauma studies (43). The use of imagination, creativity and playfulness in trauma psychotherapy has been found to improve satisfaction, self-management, self-efficacy, and post-trauma resilience (43–45).

The use of artistic COPE cards is novel part of and a major component of the SEE FAR CBT treatment for anxiety and PTSD. This treatment involves, among other things, non-verbal, psychologically safer methods to approach painful experiences by using symbols and metaphors. Arousing negatively-valenced COPE cards enable patients, playfully and therapeutically, to access deep feelings, narrate their experiences, and identify their own ways of coping with crisis, stress, and trauma. Randomly selecting cards and dealing with the associations they evoke serves a sort of virtual training in dealing with challenging situations within the safe world of image and metaphor. Relaxing positively-valenced cards can be used as calming stimuli during therapeutic session. This method offers a unique opportunity to PTSD sufferers to regain control through the use of imagination and playfulness *via* the cards (10, 16, 46).

While there are many subjective reports (by patients and therapists in the field) supporting the effectiveness of the SEE FAR CBT treatment, fewer formal, objective, controlled studies have been performed [but see (10, 11)]. Clearly more research is needed concerning the treatment as a whole and the COPE cards' use in particular. If the cards are to be employed as an experimental and/or therapeutic tool, it is imperative to ensure the existence and availability of valid, statistically supported, evidence regarding their specific effects. Cohen et al. (17) started this line of research by presenting the subjective self-reported affective states evoked by COPE therapeutic cards, and by creating standardized norms for their arousal, valence, and dominance subjective scores. The present study, relying on objective physiological measures, significantly contribute to validating the effectiveness of COPE cards in influencing the nature and degree of psychophysiological arousal which is fundamental to the therapeutic process.

Heart rate, blood pressure and HRV were measured as physiological indicators of ANS activity and responsiveness. While results concerning heart rate did not reach statistical significance, both blood pressure and HRV showed significant and reliable rapid changes in response to the associative cards. It has been reported that a state of chronic autonomic hyperarousal is involved in PTSD and that individuals with PTSD show physiological evidence of increased sympathetic dominance reflected, among other things, in increased blood pressure (47, 48) and decreased HRV (49–53). Individuals with higher resting HRV have been found to score higher on resilience questionnaires, to recover more efficiently from acute psychological stress and to be less vulnerable to the development of PTSD. It has been therefore suggested that decreased HRV might be considered as an endophenotype associated with various impairments and dysregulations characteristic of PTSD; and that HRV could serve as a valuable biomarker to monitor PTSD and treatment outcomes. An intervention that increases cardiac vagal tone may mitigate the imbalances in PTSD. Intervention studies aimed directly at increasing HRV in PTSD patients (e.g., biofeedback, mindfulness, relaxion training and, pharmacological treatment) reported promising results. Moreover, some studies have indicated that HRV reduction may predate PTSD, hence, deliberate increasing of HRV in advance in susceptible populations may reduce the development and prevalence of severe PTSD (49, 54–57).

As part of the SEE FAR CBT treatment, patients study how to reduce unpleasant internal arousal and how to gain a sense of safety and relaxedness by controlling fearful reactions. They practice somatic experience modalities and are given an explanation about the importance of being able to discharge blocked physical energy due to the trauma. “*The client is introduced to the power of fantastic reality and externalization through a process of creating an external safe space using therapeutic cards. The establishment of association between the image and the experience of relaxation and pleasantness assists the client during the therapy whenever anxiety prevails and the need for stress reduction arises, through focusing on the card, returning to the external, internalized safe space and then, returning to the therapeutic process*” (10). The current findings greatly support the use of COPE cards as an effective tool for controlling psychophysiological arousal.

Thinking beyond the scope of the present study, we suggest that HRV in particular is most sensitive and reliable measure of moment-to-moment changes in arousal, and that it may be used not just for research purposes but also in practical clinical settings. HRV has been shown high reliability as a non-invasive test for quantitative assessment of cardiovascular autonomic regulatory responses. The HRV assessment device has many advantages including portability, low-cost wearable sensors, easy application and user-friendly interfaces for data extraction and visualizations. In recent years, using biofeedback as a method to increase HRV has become increasingly popular among psychotherapists. It has been suggested that HRV biofeedback has widespread beneficial effects and that it improves symptoms and functioning in many areas, both in the normal and pathological ranges (58).

In the interpretation of our results a few considerations need to be taken into account. The current study was performed on undergraduate students without known prior history of neurological or psychiatric disorders, or traumatic experiences. Although the COPE cards were developed for therapeutic purposes, the present study does not deal with their direct therapeutic effect, but aims, following Cohen's study (17) which was also done on normative students, to test and characterize, through objective physiological measures, their ability to influence emotional arousal. Based on our experience in the Community Stress Prevention Center's clinic (www.icspc.org) and following the many reports on the impact of the exposure to the cards on clients we opted to start and examine the physiological impact of the cards on normal population as not to aggravate clients' condition when they are not in treatment. Moreover, we suggest that the cards may be used not only for therapeutic purposes, but also as an effective research tool in the general field of emotion and psychophysiological arousal, with the advantage of stimuli being less realistic and more artistic/projective/associative compared to more traditional emotionally evocative databases such as the international affective picture system [IAPS; (37)], the Geneva affective picture database [GAPED; (59)], and the Nencki affective picture system (60). For this end, it is worthwhile to examine COPE cards characteristics in normative sample. Obviously, future studies may seek to examine subjective and physiological responses to COPE cards in clinical samples, and especially among PTSD sufferers, and to compare the results with those of the current study. It is our intention to follow this study with patients in a different design and under supportive/therapeutic environment. In addition, to further examine and validate the differential effects of the various COPE cards, it would be beneficial to evaluate neurological brain responses to them by, for example, using the event-related potentials (ERP) method.

The current study was conducted on young adults in a relatively homogeneous sample in terms of age, education and ethnicity. Women made up about 62% of the total sample. It would be of importance to examine potential interactions between COPE cards effects and other variables such as age, education level, and gender. Finally, while participants were instructed to reach a subjective degree of at least 7 (on a scale of 0–10) of emotional and physical negative arousal (at the arousal phase), and then to relax to the best of their ability (at the relaxion phase), we did not record their specific subjective feelings of distress/relaxation. Such subjective assessments of emotional state may be used to examine correlations with the physiological responses.

To summarize, this study gives further support to the effect imaginal projective cards have on arousal and relaxedness thus offering a further validation to their use (beyond the classical interpretative use) in clinical and experimental settings. It also expands on the more traditional experimental sets of emotional stimuli such as the IAPS. The current study offers an indication of psychophysiological impact of the therapeutic cards and thus support their direct and indirect impact on 'emotional experience which is fundamental to the therapeutic process when treating anxiety and psychotrauma aiming at desensitization of the emotions the traumatic memory raise.

## Data availability statement

The raw data supporting the conclusions of this article will be made available by the corresponding authors on reasonable request.

## Ethics statement

The studies involving human participants were reviewed and approved by Tel-Hai College Review Board. The patients/participants provided their written informed consent to participate in this study.

## Author contributions

SR and ML contributed to conception and design of the study. SR organized the database, performed the statistical analysis, and wrote the first draft of the manuscript. All authors contributed to manuscript revision, read, and approved the submitted version.

## Funding

This study was supported by the Tel-Hai College research authority.

## Conflict of interest

The authors declare that the research was conducted in the absence of any commercial or financial relationships that could be construed as a potential conflict of interest.

## Publisher's note

All claims expressed in this article are solely those of the authors and do not necessarily represent those of their affiliated organizations, or those of the publisher, the editors and the reviewers. Any product that may be evaluated in this article, or claim that may be made by its manufacturer, is not guaranteed or endorsed by the publisher.

## Footnotes

^1^No correlations were found between the physiological measures and trait anxiety [STAI; (34)], fantastic reality/imagination ability [FRAME; (35)], or trait emotional intelligence [TEIQue-SF; (36)].
